# Physiology

**Published:** 1838-07

**Authors:** 


### PART FOURTH.

### Selections from the British Journals.

(FOR THE QUARTER ENDING MAY 31, 1838.)

---

### PHYSIOLOGY.

### *Case of Paraplegia.* By Wm. Elliott, m.d., Carlisle.

This is an ordinary example of Pott's disease. The only point of any interest in it is the well-attested fact of motion being produced in one of the paralysed limbs by irritation applied to the part, and not exciting any previous sensation.

"On pricking, scratching, or pinching the skin of the left foot, indications of sensation of pain occur, and the girl asserts she feels pain: at the same time irregular movements of the left toes, left foot, and left leg, and sometimes of the thigh also, follow, over which she has no control. But, on irritating the integuments of the right foot in a similar manner, there is not, even when blood is drawn, the slightest indication of pain: the girl asserts she feels none; and irregular muscular movements of the right toes, foot, leg, and thigh are produced. These movements are sometimes those of flexion, sometimes those of extension, and are not those producing a withdrawal of the limb, or such as would appear the natural result of the sensation of pain in the part irritated."

*Lancet. April* 14, 1838.

